# ASO Author Reflections: Clinical Staging of Early Gastric Cancer—We’re Still Missing the Mark

**DOI:** 10.1245/s10434-026-20190-6

**Published:** 2026-07-17

**Authors:** Alexandra Adams, Haejin In

**Affiliations:** 1 Division of Surgical Oncology, Rutgers Cancer Institute of New Jersey, New Brunswick, NJ USA; 2 Department of Health, Behavior, and Policy, Rutgers School of Public Health, Piscataway, NJ USA

## Past

In the era of multidisciplinary cancer care, accurate clinical staging is paramount for selecting the appropriate treatment and sequence. In gastric cancer (GC), treatment of clinical stage I patients is unique from later stages, as upfront surgical resection is standard of care, whereas higher-stage disease is increasingly treated with neoadjuvant or perioperative systemic chemotherapy.^1^ Unfortunately, our ability to clinically stage early GC remains imperfect. More than one-third of patients with stage I GC are understaged, and this rate has remained largely unchanged for nearly a decade.^2,3^ As a result, many patients proceed directly to surgery despite harboring more advanced disease, potentially missing the opportunity to receive contemporary multimodal therapies that may improve outcomes.^4^ We sought to identify factors associated with an increased risk of understaging among patients with presumed early-stage GC.

## Present

In this NCDB analysis, we found that 36% of patients with clinical stage I GC were understaged on final pathologic assessment.^3^ Increasing tumor size and higher grade were independently associated with a greater risk of understaging, and the risk compounded when these risks coexisted. These findings identify readily available clinical characteristics that may help recognize patients at increased risk for occult higher-stage disease before treatment selection is finalized.

## Future

Despite advances in endoscopic technology, clinical staging of early gastric cancer remains imperfect. As treatment paradigms evolve, the consequences of understaging have become increasingly significant, as patients with occult higher-stage disease may proceed directly to surgery and miss the opportunity to receive effective neoadjuvant or perioperative therapies.

While tumor size is not routinely measured and reported during staging endoscopy, we would argue that it should be considered a standard component of clinical assessment given its strong association with occult higher-stage disease. Incorporating tumor size and differentiation into clinical staging algorithms may improve risk stratification among patients with presumed early-stage gastric cancer, identify those at higher risk for understaging, and facilitate more informed treatment selection.
